# Evaluation of an ultra-portable X-ray system with automated interpretation for tuberculosis active case finding in carceral settings: a diagnostic test accuracy study

**DOI:** 10.21203/rs.3.rs-5578367/v1

**Published:** 2025-04-07

**Authors:** Argita D. Salindri, José V. B. Bampi, Caroline Busatto, Alessandra M. da Silva, Andrea da Silva Santos, Isabella B. Gonçalves, Thais O. Gonçalves, Eunice A. T. Cunha, Daniel Tsuha, Everton Lemos, Roberto D. Oliveira, Mariana Croda, Jason R. Andrews, Julio Croda

**Affiliations:** Stanford University School of Medicine; Federal University of Mato Grosso do Sul, Mato Grosso do Sul; Federal University of Mato Grosso do Sul, Mato Grosso do Sul; Federal University of Mato Grosso do Sul, Mato Grosso do Sul; Federal University of Grande Dourados; Federal University of Mato Grosso do Sul, Mato Grosso do Sul; Central Laboratory of Mato Gross do Sul; Central Laboratory of Mato Gross do Sul; Federal University of Mato Grosso do Sul, Mato Grosso do Sul; Federal University of Mato Grosso do Sul, Mato Grosso do Sul; Federal University of Grande Dourados; Federal University of Mato Grosso do Sul, Mato Grosso do Sul; Stanford University School of Medicine; Federal University of Mato Grosso do Sul, Mato Grosso do Sul

**Keywords:** active case finding, persons deprived of liberty, ultra-portable X-ray, LunitTB

## Abstract

**Background:**

The World Health Organization recommends systematic active case finding for tuberculosis (TB) among high-risk population including incarcerated individuals; however, many prisons lack screening capacity. In this study, we aimed to evaluate the diagnostic performance of an ultra-portable digital chest radiography system paired with LunitTB, an automated interpretation algorithm, to detect TB disease.

**Methods:**

We performed a diagnostic test accuracy study using data collected for a prospective active case finding study for TB in a Brazilian prison from February 2023 through May 2024. Eligible individuals included adults (≥18 years) without a TB history in the past two years. A Fujifilm Digital Radiography (FDR) Xair paired with LunitTB algorithm (version v3.1.5.1) system was used to screen consented individuals for TB disease irrespective of their TB symptoms. Area under curve (AUC) and 95% confidence intervals (CI) were estimated to determine the accuracy of FDR Xair and LunitTB interpretation when compared to a rigorous microbiologic reference standard.

**Results:**

We screened a total of 3409 individuals for TB disease as part of our active TB case finding study, and 3399 (99.7%) met our eligibility criteria for the diagnostic test accuracy study. TB prevalence was 4.1% (139/3399, 95%CI 3.5–4.8%). The AUC for FDR Xair and LunitTB interpretation was 0.89 (0.86–0.93). The accuracy of FDR Xair and LunitTB interpretation among those with any TB symptoms was significantly higher (AUC = 0.93, 95%CI 0.90–0.97) compared to those without TB symptoms (AUC = 0.87, 95%CI 0.81–0.92) (DeLong p = 0.033).

**Conclusions:**

The FDR Xair and LunitTB interpretation enabled us to screen persons deprived of liberty rapidly, with a high diagnostic accuracy especially among those reported any TB symptoms.

## INTRODUCTION

Globally, persons deprived of liberty (PDL) have exceedingly high risk of tuberculosis (TB); especially in South American countries where the incidence rate was estimated to be nearly 27-fold than the general population. [1] In 2021, the World Health Organization (WHO) recommended systematic screening of PDL for TB disease. [2] However, implementation of this recommendation has been limited in low- and middle-income countries, as many carceral institutions lack resources and equipment for systematic TB screening. There is a critical need to identify efficient strategies for TB active case finding that can be scaled in carceral settings in high TB burden countries.

In recent years, there have major advances in the use of artificial intelligence to interpret chest X-ray (CXR) images for TB screening, with promising results; however, the majority of published studies were conducted among symptomatic individuals presenting to clinical settings. [3] Recent technological development in digital X-ray imaging and display have provided an opportunity to bringing care outside of healthcare facilities through portable X-ray devices. [4] The use of portable X-ray system paired with an automated X-ray interpretations was recently shown to be effective for community-based TB screening in an evaluation in Nigeria. [5] Furthermore, a recent systematic review aiming to evaluate the performance of different CXR paired with AI software reported a pooled sensitivity of 94% (89–96%) and a pooled specificity of 95% (91–97%) in model-development studies (i.e., non-trial studies). [6] However, the accuracy of ultra-portable radiography with automated interpretation compared with a rigorous microbiologic reference standard remains unclear. Thus, we aimed to evaluate the diagnostic performance of an ultra-portable X-ray device and automated interpretation system as a screening test for active TB case finding efforts among the prison population.

## METHODS

### Study population, design, and setting

We conducted a diagnostic test accuracy study using data collected for a prospective TB active case finding study in a large male prison in the state of Mato Grosso do Sul, Brazil, from February, 2023 through May, 2024. For the active TB case finding study, all adult PDL (^3^18 years) were approached; and after obtaining informed consent, study staff administered structured demographic and clinical questionnaires. All study participants were then asked to provide a spot sputum sample, which was divided for a) pooled GeneXpert Ultra (Xpert) with a pool size of eight as previously described [7, 8] and b) Ogawa culture testing. By the end of enrollment day, all sputum samples were transported to the local public health reference laboratory where all microbiological works was performed. After sputum collection, a posterior-anterior chest X-ray was obtained using Fujifilm Digital Radiography (FDR) Xair XD2000 PX for all participants irrespective of TB symptoms. FDR Xair is a lightweight digital radiography system. X-ray images were scored using LunitTB algorithms v3.1.5.1 developed by Lunit (Seoul, South Korea), which provides a numerical TB risk score between 0 and 100. For this diagnostic test accuracy study, eligible participants included consented individuals with no history of TB treatment in the past two years.

### Study measures, definitions, and statistical analysis

Demographic characteristics and clinical data were collected using a structured study questionnaire **(Supplemental Material 1)** developed for the present study’s purposes and recorded using REDCap [9] online data capture tools hosted at the Federal University of Mato Grosso do Sul. Collected information included age, highest education attainment, incarceration history, previous TB history, TB symptoms at the time of TB screening, and behavioral risk factors (e.g., smoking and drug use). We categorized smoking status into “current smoker” and “never/former smoker.” We define any drug use if study participants reported any drug use in the past 12-months period.

Individuals with a positive Xpert result at screening had an Xpert confirmatory test done; thus, we defined our study outcome, TB disease, if individuals had a) a positive culture result, b) two positive Xpert test results, or c) one positive and one trace Xpert results. [10]

### Statistical Analyses

We used chi-square and Fisher’s exact tests to assess bivariate associations between participants’ characteristics and TB status. We used Wilcoxon rank sum test to compare the median of LunitTB scores among individuals with and without TB disease. We then estimated the area under the receiver operating characteristic (ROC) curve (AUC) and the 95% confidence interval (CI) to quantify the accuracy of FDR Xair and LunitTB screening system as a quantitative diagnostic for TB disease. We performed sensitivity analyses to evaluate the performance of FDR Xair and LunitTB screening system when different definitions were used to define TB (i.e., a positive result on both culture and Xpert, and a positive result on either culture or Xpert). We also calculated sensitivity at the LunitTB threshold that achieved 70% specificity (LunitTB score=42.8), and specificity at 90% sensitivity (LunitTB score=32.7), corresponding to the WHO screening benchmarks. [2] We used DeLong test [11] to compare areas under correlated ROC curves among key sub-populations. We compared LunitTB scores and evaluated sensitivity according to the semi-quantitative Xpert result, as a measure of bacillary burden.

### Ethics approval and consent to participate

The study was approved by the Research Ethics Committee of the Federal University of Mato Grosso do Sul (#5.730.361), the National Research Ethics Committee of Brazil (CONEP) (#5.899.470), and the Institutional Review Boards (IRB) at Stanford University (IRB#67287). All study participants provided written informed consent prior to study participation and study procedures were performed in accordance with relevant guidelines and regulations.

## RESULTS

### Characteristics of study participants and TB prevalence

**Figure 1:**
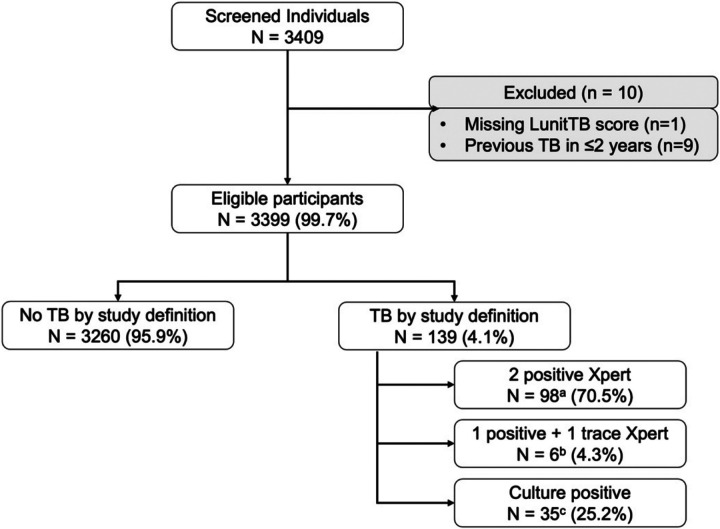
Study participants selection and tuberculosis case definition ^a^ Of those defines as TB by 2 positive Xpert, 72 (73.5%) had a positive culture result while 22 (22.4%) had a negative culture results, and 4 (4.1%) others had a missing culture results (e.g., contaminated) ^b^ Of those defines as TB by 1 positive and 1 trace results on Xpert, all (6/6, 100%) had a negative culture result ^c^ Of those defined as TB by a positive culture result, 28 (80.0%) had 1 positive Xpert result while 7 (20.0%) others had negative Xpert results

We screened 3409 individuals for TB disease as part of our active TB case finding study in 85 working days, for a median of 40 individuals screened per day and a maximum of 96. Among these, 3408 (99.9%) had LunitTB score and TB status information available, nine of whom had a TB episode within two years prior to study enrollment and were excluded, leaving 3399 (99.7%) individuals included in the final analyses (Fig. 1). Characteristics of individuals screened are provided in Table 1.

**Figure 2:**
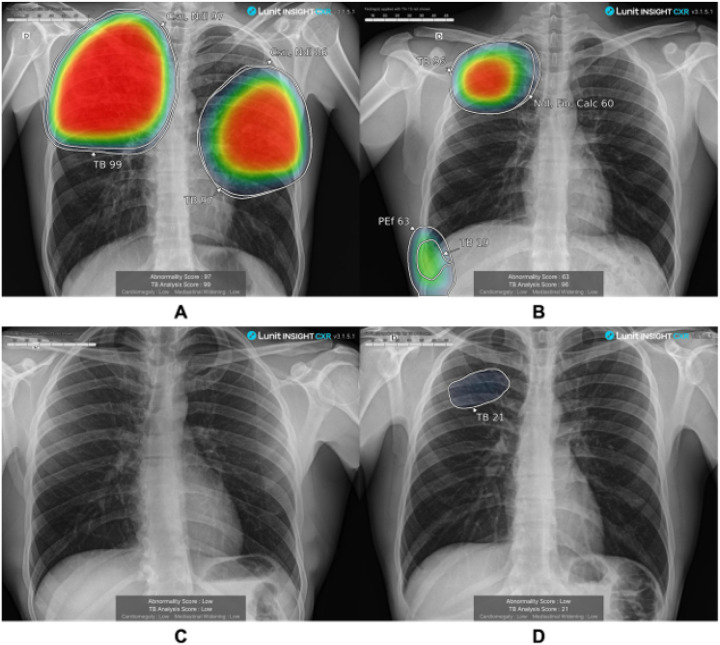
Chest X-ray images produced with FDR Xair and scored with LunitTB for two individuals with tuberculosis (A and B) and two individuals without tuberculosis (C and D)

Overall, TB prevalence was 4.1% (139/3399, 95%CI 3.5–4.8) (Table 1). Compared to individuals without TB, those with TB were more likely to have a history of TB treatment > 2 years prior to study enrollment, report at least one TB symptom, and currently smoke tobacco products (p < 0.05). The median LunitTB score was significantly higher among individuals with TB (median = 96.7, interquartile range [IQR] 90.0–98.4) compared to those without TB (median = 29.0, IQR 15.0–49.1, p-value < 0.001). Samples of scored chest X-ray images from individuals with and without TB disease are provided in Fig. 2.

### Diagnostic accuracy of the FDR Xair and LunitTB interpretation

**Figure 3:**
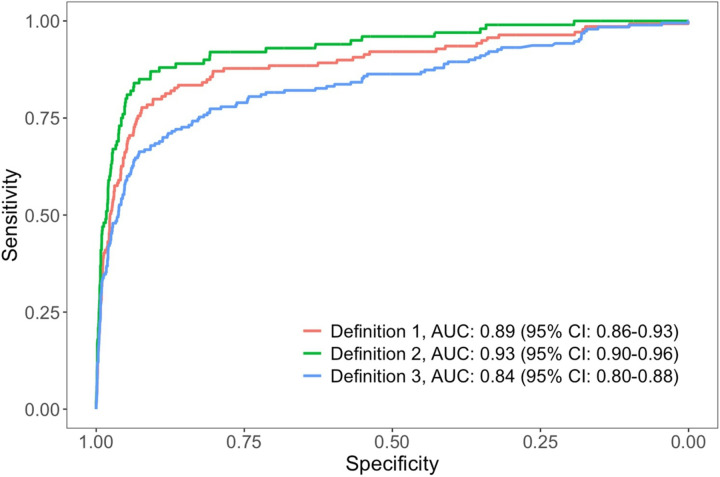
Receiver operating characteristic (ROC) curves and area under the curve (AUC) for LunitTB as a screening test for active TB case finding efforts among persons deprived of liberty in Mato Grosso do Sul, Brazil, February 2023 – May 2024 (N=3,399) Definition 1: a positive culture OR two positive Xpert OR one positive Xpert with an Xpert trace Definition 2: a positive culture AND at least one positive Xpert Definition 3: a positive culture OR at least one positive Xpert

Overall, the area under curve (AUC) for TB prediction using FDR Xair and LunitTB interpretation was 0.89 (95%CI 0.86–0.93) (Fig. 3). Using a positive Xpert and a positive culture to define TB, the AUC was improved to 0.93 (95%CI 0.90–0.96) (p = 0.076). The AUC was lower when using a positive Xpert or a positive culture to define TB (AUC = 0.84, 95%CI 0.80–0.88) (p = 0.048).

**Figure 4.**
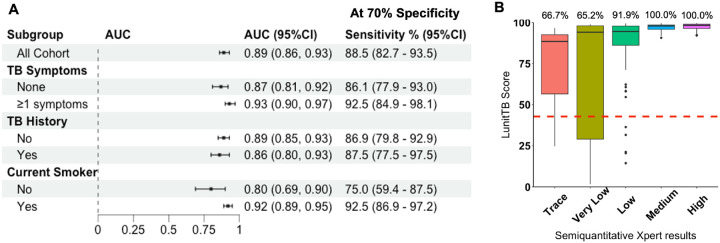
Performance of Fujifilm FDR Xair-LunitTB as a quantitative TB diagnostic among persons deprived of liberty in Brazil. The diagnostic performance of Fujifilm FDR Xair-LunitTB system was expressed as the area under receiver operating characteristic curves (AUC) and 95% confidence interval, estimated for all study participants and sub-groups within the study prison (A). We also described the LunitTB score distribution according to the bacterial load among individuals with positive culture or Xpert results, with a red dashed line to indicate the threshold where specificity is at 70% (LunitTB score=42.8) and the percentages on top of each box plot represent the proportion of individuals with TB that had LunitTB score >42.8 (B).

The diagnostic accuracy among those with any TB symptoms (AUC = 0.93, 95%CI 0.90–0.97) was significantly higher compared to those without TB symptoms (AUC = 0.87, 95%CI 0.81–0.92) (p = 0.033) (Fig. 4A). Similarly, diagnostic accuracy was significantly higher among current smokers (AUC = 0.92, 95%CI 0.89–0.95) compared to never/former smokers (AUC = 0.80, 95%CI 0.69–0.90) (p = 0.028). The diagnostic accuracy was similar among individuals with or without history of TB episode > 2 years prior to study enrollment (p = 0.552).

Among individuals with any TB symptoms, at 70% specificity, the screening system met the WHO target product profile (TPP) [2] thresholds for a screening test with a sensitivity of 92.5% (95%CI 84.9–98.1) (Fig. 4A). Similarly, among individuals with any TB symptoms, at 90% sensitivity, the FDR Xair and LunitTB interpretation met the WHO thresholds for a screening test with a specificity of 89.1% (95%CI 52.6–94.5) (data not shown).

### Considerations to use FDR Xair and LunitTB interpretation in an active TB case finding effort

Nearly a third (32.3%, 1099/3399) of study participants had LunitTB score > 42.8 (data not shown). Performing individual Xpert tests among those with LunitTB score > 42.8, we would have used 68% fewer Xpert cartridges (i.e., compared to performing individual Xpert tests among all study participants; 1099 vs. 3399 cartridges) while maintaining sensitivity to identify a high proportion (123/139, 88.5%) of individuals with TB disease (Fig. 4A). Among the 16 individuals with TB and LunitTB score ≤42.8 that we would have missed without individual Xpert testing, 2 had positive culture and negative Xpert results, while 14 others had either low (n = 5), very low (n = 8) or trace Xpert (n = 1) test results (Fig. 4B). LunitTB scores were strongly correlated with Xpert semi-quantitative load (Spearman’s ρ = 0.387, p < 0.001), and all 47 (100%) study participants with medium (n = 23) or high (n = 24) bacterial load were identified by using the 42.8 LunitTB score threshold (Fig. 4B).

Among our study participants, there were 578 (17.0%, 578/3399) individuals who reported at least one TB symptoms, 220 (38.1%) of whom had LunitTB score > 42.8. Performing individual Xpert and/or culture tests (i.e., microbiologic reference standard) among those with LunitTB score > 42.8 and ≥1 TB symptoms, we would have identified approximately one-third (35.3%, 49/139) of all TB cases per our study definition (data not shown). Symptoms screening alone with Xpert confirmation would have detected 38.1% (95%CI 30.3–46.4%, 53/139) of TB cases.

## DISCUSSION

Overall, the FDR Xair and LunitTB interpretation enabled efficient TB active case finding to be performed among PDL and met the WHO TPP benchmarks, achieving particularly high accuracy among individuals with any TB symptoms. Furthermore, the FDR-Xair-LunitTB system was sensitive in identifying PDL with medium/high bacterial load, who may be more likely to transmit *Mtb*. Incorporating FDR Xair and LunitTB interpretation in active case finding programs in prisons could reduce the number of Xpert cartridges used while identifying individuals at highest risk for morbidity and transmission.

While the WHO recommends use of chest radiography for TB active case finding, many institutional correctional or detention facilities in high TB burden countries do not have X-ray equipment or have equipment that are not functioning [12]. Repairs are often delayed or not performed due to need for technical personnel to travel to prisons and obtained security clearances. We previously used a mobile X-ray machine installed on a container truck for TB screening in prisons, finding high accuracy of its use combined with automated interpretation for TB screening [13]. However, this approach required transportation of PDL from the cell blocks to the exterior courtyard of the prison, which required substantial security personnel time and slowed the pace of screening. The ultra-portable, battery-powered, FDR Xair–LunitTB system enabled screening to be done within the cell blocks, achieving more rapid screening, and can be transported between prisons to increase access to screening while containing costs. It requires low to no constructions costs, produced high quality images [14], can be repaired off-site, and has acceptable radiation risks that can be enhanced with portable lead curtains [15].

The accuracy of the Xair–LunitTB screening system was higher among individuals with TB symptoms. Individuals with symptomatic TB were more likely to have higher Xpert semiquantitative loads than those with subclinical TB, and we believe the greater accuracy of the radiographic screening reflects greater sensitivity in more advanced TB disease. Screening sensitivity was moderately high (86% at 70% specificity) among those without symptoms, but this fell short of WHO TPP benchmarks. It is critical to note that only one-third of our study participants reported ≥1 TB symptoms. The use of the ultra-portable X-ray with LunitTB interpretation among symptomatic individuals would have only detected one-third of TB cases in the prison. As individuals with subclinical TB (i.e., asymptomatic) may play an important role in transmission, these findings suggest a need to further evaluate the consideration of lowering the threshold especially among this sub-group or to identify alternative tools to improve screening sensitivity.

Our finding also suggested that approximately one out of nine (~ 11.5%) TB cases in our study would not have been identified using CXR and LunitTB system alone. The use of CXR and LunitTB in combination with other screening strategies (e.g., TB symptoms assessment and Xpert confirmation) may improve screening yields as documented in our previous study. [16] Future studies should also consider evaluating the added value of periodically rescreening individuals with abnormal CXR findings at baseline screening as these individuals may be at greater risks to develop TB disease. [17]

Our study has several limitations. First, our study was conducted in a single maximum-security prison in Brazil, and the results, may not be generalizable to other settings with different demographic and epidemiologic characteristics. Specifically, the LunitTB score thresholds identified in this population should not be extrapolated to other study populations (e.g., household contacts) as TB risk factors (e.g., smoking, poor ventilations) may be more common among PDL. [18] Second, we used spot sputum samples for Xpert and/or culture testing, which may lead to misclassification of TB status. Third, we did not perform a proper costing analysis (i.e., cost-effectiveness analysis) to measure how much screening costs were saved by using the ultra-portable X-ray and LunitTB interpretation compared to other screening strategies. However, our previous work suggested that screening strategies which utilized the combination of symptoms screening, chest X-ray with an automated interpretation, and Xpert confirmation had a slightly higher yield (76% vs. 74% when using sputum Xpert for all participants) with an average cost per case diagnosed of US$395. [16] Fourth, we did not perform a validity study to compare the AI reading with a radiologist reading to interpret CXR images. Fifth, our study was conducted in settings where HIV prevalence was low (~ 1%) [19]. Thus, we did not do any stratifications according to HIV status due to the small sample size (n = 14) as it will be challenging to make any inferences on HIV-related findings.

## CONCLUSION

In conclusion, the FDR Xair and LunitTB interpretation enabled us to screen PDL rapidly, with a high diagnostic accuracy especially among individuals reporting any TB symptoms. Further studies to assess the performance of FDR Xair and LunitTB interpretation in other settings are still warranted.

## Figures and Tables

**Table 1.** Characteristics and risk factors for prevalent tuberculosis among persons deprived of liberty in Mato Grosso do Sul, Brazil, February 2023 – May 2024 (N=3,399)

Characteristics	Total N = 3399	Tuberculosis Status*		p- values^†^
		No	Yes	
		N (%) = 3260 (95.9)	N (%) = 139 (4.1)	
Median age, years (IQR)	31 (26 – 37)	31 (26 – 37)	30 (26 – 36)	0.4985
Race				
White	634 (18.7)	617 (18.9)	17 (12.2)	**0.032** ^§^
Black	292 (8.6)	279 (8.6)	13 (9.4)	
Mixed	2466 (72.6)	2358 (72.3)	108 (77.7)	
Asian	4 (0.1)	4 (0.1)	0 (0.0)	
Indigenous	3 (0.1)	2 (0.1)	1 (0.7)	
Education attainment				
Did not complete high school	2908 (85.6)	2781 (85.3)	127 (91.4)	**0.047**
Completed high school	491 (14.4)	479 (14.7)	12 (8.6)	
Previously incarcerated	2837 (83.5)	2714 (83.3)	123 (88.5)	0.104
Smoking status				
Never/former smoker	1320 (38.8)	1288 (39.5)	32 (23.0)	**<0.001**
Current smoker	2079 (61.2)	1972 (60.5)	107 (77.0)	
Any drug use	2286 (67.3)	2183 (67.0)	103 (74.1)	0.079
Marijuana	2072/2286 (90.6)	1978/2183 (90.6)	94/103 (91.3)	0.824
Cocaine			52/103 (50.5)	0.709
Crack	1113/2286 (48.7)	1061/2183 (48.6)	2/103 (1.9)	1.000^§^
Heroin	55/2286 (2.4)	53/2183 (2.4)	0/103 (0.0)	1.000^§^
Glue and/or other solvents	10/2286 (0.4)	10/2183 (0.5)	1/103 (1.0)	0.565^§^
Pasta-based	18/2286 (0.8)	17/2183 (0.8)	3/103 (2.9)	
Hashish	62/2286 (2.7)	59/2183 (2.7)	0/103 (0.0)	0.757^§^
Injectables	27/2286 (1.2)	27/2183 (1.2)	0/103 (0.0)	0.631^§^
	2/2286 (0.1)	2/2183 (0.1)		1.000^§^
Previous TB^a^	574 (16.9)	534 (16.4)	40 (28.8)	**<0.001**
Any TB symptoms	578 (17.0)	525 (16.1)	53 (38.1)	**<0.001**
Cough	474/578 (82.0)	423/525 (80.6)	51/53 (96.2)	**0.005**
Productive cough	375/578 (64.9)	333/525 (63.4)	42/53 (79.2)	**0.022**
Blood-stained sputum	35/578 (6.1)	28/525 (5.3)	7/53 (13.2)	**0.012** ^§^
Fever	170/578 (29.4)	146/525 (27.8)	24/53 (45.3)	**0.008**
Loss of appetite	86/578 (14.9)	71/525 (13.5)	15/53 (28.3)	**0.004**
Weight loss	145/578 (25.1)	126/525 (24.0)	19/53 (35.8)	0.058
Night sweats	91/578 (15.7)	77/525 (14.7)	14/53 (26.4)	**0.025**
Chest pain	215/578 (37.2)	183/525 (34.9)	32/53 (60.4)	**<0.001**
Difficulty in breathing	182/578 (31.5)	158/525 (30.1)	24/53 (45.3)	**0.023**
Contact with TB-sick person (N=3398)	2044 (60.2)	1962 (60.2)	82 (59.0)	0.775
Yes, 1 – 3 times per week	146/2044 (7.1)	142/1961 (7.2)	4/83 (4.9)	0.608
Yes, 4 – 6 times per week	42/2044 (2.1)	41/1961 (2.1)	1/83 (1.2)	
Yes, everyday	1856/2044 (90.8)	1779/1961 (90.7)	77/83 (93.9)	
LunitTB score, median (IQR)	29.9 (15.0 – 53.4)	29.0 (15.0 – 49.1)	96.7 (89.9 – 98.4)	**<0.001** ^‡^

* Tuberculosis case was defined by a positive culture or two positive GeneXpert or one positive and one trace GeneXpert results

† p-values from Chi-square tests, unless indicated otherwise

‡ p-values from Wilcoxon rank-sum tests

§ p-values from Fisher’s exact tests

a Previous TB episode dated >2 years prior to study enrollment

Abbreviations

IQR – interquartile range; TB – tuberculosis

**Bold** indicates that the finding was statistically significant at a=0.05

## Abbreviations

AUC: Area under the curve
CI: Confidence interval
FDR: Fujifilm digital radiography
IQR: Interquartile range
PDL: Persons deprived of Liberty
ROC: Receiver operating characteristics
TB: Tuberculosis
TPP: Target Product Profile
WHO: World Health Organization
